# Insights into the synthesis, engineering, and functions of microbial pigments in *Deinococcus* bacteria

**DOI:** 10.3389/fmicb.2024.1447785

**Published:** 2024-07-25

**Authors:** Yuxian Wang, Jiayu Liu, Yuanyang Yi, Liying Zhu, Minghui Liu, Zhidong Zhang, Qiong Xie, Ling Jiang

**Affiliations:** ^1^College of Food Science and Light Industry, Nanjing Tech University, Nanjing, China; ^2^Institute of Applied Microbiology, Xinjiang Academy of Agricultural Sciences/ Xinjiang Key Laboratory of Special Environmental Microbiology, Urumqi, China; ^3^College of Life Sciences, Xinjiang Normal University, Urumqi, China; ^4^School of Chemistry and Molecular Engineering, Nanjing Tech University, Nanjing, China; ^5^School of Pharmaceutical Sciences, Nanjing Tech University, Nanjing, China; ^6^China Astronaut Research and Training Center, Beijing, China; ^7^State Key Laboratory of Materials-Oriented Chemical Engineering, College of Food Science and Light Industry, Nanjing Tech University, Nanjing, China

**Keywords:** *Deinococcus*, carotenoid, deinoxanthin, metabolic engineering, antioxidation

## Abstract

The ability of *Deinococcus* bacteria to survive in harsh environments, such as high radiation, extreme temperature, and dryness, is mainly attributed to the generation of unique pigments, especially carotenoids. Although the limited number of natural pigments produced by these bacteria restricts their industrial potential, metabolic engineering and synthetic biology can significantly increase pigment yield and expand their application prospects. In this study, we review the properties, biosynthetic pathways, and functions of key enzymes and genes related to these pigments and explore strategies for improving pigment production through gene editing and optimization of culture conditions. Additionally, studies have highlighted the unique role of these pigments in antioxidant activity and radiation resistance, particularly emphasizing the critical functions of deinoxanthin in *D. radiodurans*. In the future, *Deinococcus* bacterial pigments will have broad application prospects in the food industry, drug production, and space exploration, where they can serve as radiation indicators and natural antioxidants to protect astronauts’ health during long-term space flights.

## 1 Introduction

Color plays a pivotal role in food production and processing, serving to facilitate product identification, preserve sensory quality, enhance original colors, preserve nutritional value, and improve consumer acceptance (Ghosh et al., 2024). Therefore, for many foods, adding food colorants prior to final packaging or consumption is an indispensable component of their formulation. Chemically synthesized colorants have gained popularity as they offer advantages such as cost-effectiveness in production, high tinctorial strength, and robust chemical stability (Sen et al., 2019). However, concerns regarding potential adverse effects associated with synthetic colorants, including hyperactivity in children, allergenicity, toxicological issues, and carcinogenicity problems, have prompted the prohibition of several synthetic food colorants (Sen et al., 2019). This has subsequently led to a transition from the use of synthetic food colorants to natural ones. Natural pigments derived from plants, microorganisms, minerals, insects, and marine organisms present promising alternatives to synthetic food colorants (Park et al., 2020). Among them, microbes produce a variety of pigments that can be used as additives, antioxidants, color intensifiers, and functional food ingredients (Rajendran et al., 2023).

Bacterial pigments are a diverse class of compounds produced by microorganisms during their physiological processes and are widely present in nature (Venil et al., 2013). These pigments not only play important roles in the ecological adaptation of bacteria but also have a variety of potential industrial applications (Orlandi et al., 2022). Bacterial pigments include carotenoids, melanin, phycobilin, and flavonoids. Among them, carotenoids have attracted much attention due to their diverse physiological functions and unique physicochemical properties (Celedón and Díaz, 2021). Carotenoids are a class of polyene compounds composed of 40 or 50 carbon atoms that are widely found in plants, algae, fungi, and bacteria (Maoka, 2020; Giani et al., 2024). They not only give organisms bright colors but also possess various biological functions such as antioxidant activity, photoprotection, and immune regulation (Esteban et al., 2015; Maoka, 2020; Flieger et al., 2024). In bacteria, the synthesis pathways and functions of carotenoids are diverse. They involve the synergistic action of multiple genes and enzymes, which makes the study of bacterial carotenoids one of the hotspots (Liang et al., 2018; Wurtzel, 2019).

Currently, extremophiles have emerged as promising bioresources for the production of diverse pigments due to their involvement in crucial cellular functions necessary for survival in extreme habitats (Jeong et al., 2021). The phylum *Deinococcus–Thermus* is one group of extremophiles that possesses the ability to synthesize carotenoids, resulting in the formation of colonies exhibiting red, orange, and pink colors (Tian and Hua, 2010). Compared to other carotenoids, those produced by *Deinococcus* species demonstrate excellent scavenging activity against singlet oxygen and hydrogen peroxide, thereby exhibiting enhanced antioxidant effects (Jeong et al., 2021). Consequently, they have garnered attention as the next-generation organic compounds and hold great potential as natural ingredients in food supplements.

Here, we review the bacterial pigments known as carotenoids, which are found in *Deinococcus* bacteria either naturally or through genetic engineering. We introduce their physicochemical properties and applications in various fields. The biosynthetic pathway of carotenoids is analyzed, and the key enzymes involved in the pathway are introduced individually. In addition, we discuss metabolic engineering techniques used to enhance carotenoid production in *Deinococcus,* including genetic engineering and modifications to culture conditions aimed at improving yield. We also explore gene editing technology for producing other types of carotenoids and heterologous expression of pathway genes for carotenoid production. Finally, the roles of these carotenoids in conferring stress resistance to *Deinococcus* bacteria were summarized, highlighting their importance in combating oxidative stress.

## 2 Extremophilic *Deinococcus* bacteria

There are numerous types of bacteria on Earth, and they exist in nearly all environments (Poli et al., 2017). Specifically, certain bacteria can even survive in extreme environments such as high temperatures, high pressures, and acidic or alkaline conditions (Shu and Huang, 2022). The radiation environment is an extreme condition because it can damage the genetic material DNA and other biological macromolecules of living organisms (Romanovskaya et al., 2002). *Deinococcus radiodurans* (*D. radiodurans*) can tolerate lethal doses of ionizing radiation and ultraviolet radiation (Nayak et al., 2021). It belongs to the *Deinococcaceae* family, and bacteria in this family play an important role in bioremediation due to their high tolerance to radiation (Basu, 2022). Most bacteria in the genus *Deinococcus* are Gram-positive but have the distinctive feature of Gram-negative bacteria, that is, the cells are coated with an outer membrane. The majority of these bacteria present positive on the Gram stain test, with a few exceptions, such as *D. grandis*. In addition, these bacteria all possess a thick sacculus characteristic of Gram-positive bacteria (Griffiths and Gupta, 2004). In terms of colony morphology, colonies belonging to this genus appear smooth, raised, and round-shaped with regular edges. Moreover, the colors of these colonies are rich and diverse, ranging from pink to red due to the unique carotenoids produced by them.

*Deinococcus radiodurans* and other bacteria of the genus *Deinococcus* are highly radioresistant and can survive in ionizing radiation at doses up to 5 kilogray (kGy). In contrast, radiation doses of a few hundred gray are sufficient to kill most known bacterial species, such as *Escherichia coli* (*E. coli*), while 5–10 gray is lethal for most vertebrates, including humans (Daly, 2012). An in-depth study of *D. radiodurans* will not only reveal the mechanism of radiation resistance in *Deinococcus* but also provide valuable insights into radiation resistance or sensitivity in other organisms. At present, most studies suggest that factors affecting the radiation resistance of bacteria mainly include the DNA repair system (Villa et al., 2021; Han et al., 2022), oxidative stress system (Han et al., 2020; Chen et al., 2021), and metabolic regulation system (Lim et al., 2019; Jeong et al., 2021). The oxidative stress system comprises non-enzymatic antioxidant systems such as carotenoids, bacillus thiol, and manganese complexes (Sun et al., 2010; Tian and Hua, 2010; Luan et al., 2014). Deinoxanthin, the major carotenoid in *D. radiodurans*, plays a crucial role in its antioxidant capacity and resistance against DNA damage (Sadowska-Bartosz and Bartosz, 2023). This carotenoid contributes significantly to cell defense systems and aids bacterial survival and proliferation in harsh environments (Günay and Kuzucu, 2023). Further studies have also revealed that *D. radiodurans* enhances its resistance to environmental stress through its carotenoid synthesis pathway, providing new insights into the environmental adaptability of microorganisms (Tapia et al., 2021).

Carotenoids produced by *Deinococcus* bacteria not only contribute to their survival in extreme environments but also have become a research hotspot due to their potential health benefits. For example, deinoxanthin produced by *D.* sp. UDEC-P1 showed inhibitory effects on certain cancer cells, suggesting the potential of these pigments as supplements for anticancer therapies (Tapia et al., 2021). In addition, owing to its excellent antioxidant properties, deinoxanthin has also been investigated as a potential ingredient in food additives and nutraceuticals (Jeong et al., 2021). Through genetic and metabolic engineering techniques, researchers have successfully enhanced the production capacity of deinoxanthin in *D. radiodurans*, laying the foundation for its commercial application.

## 3 Bacterial pigments in the genus *Deinococcus*

The colonies of most strains in the *Deinococcus* genus are orange or pink, a characteristic attributed to their ability to produce pigments. These pigments, produced by *Deinococcus*, serve diverse functions and are among the primary factors that enable these radiation-resistant bacteria to defend themselves against intense radiation and extremely high temperatures.

### 3.1 Deinoxanthin

#### 3.1.1 Physicochemical properties of deinoxanthin

**Figure 1:**
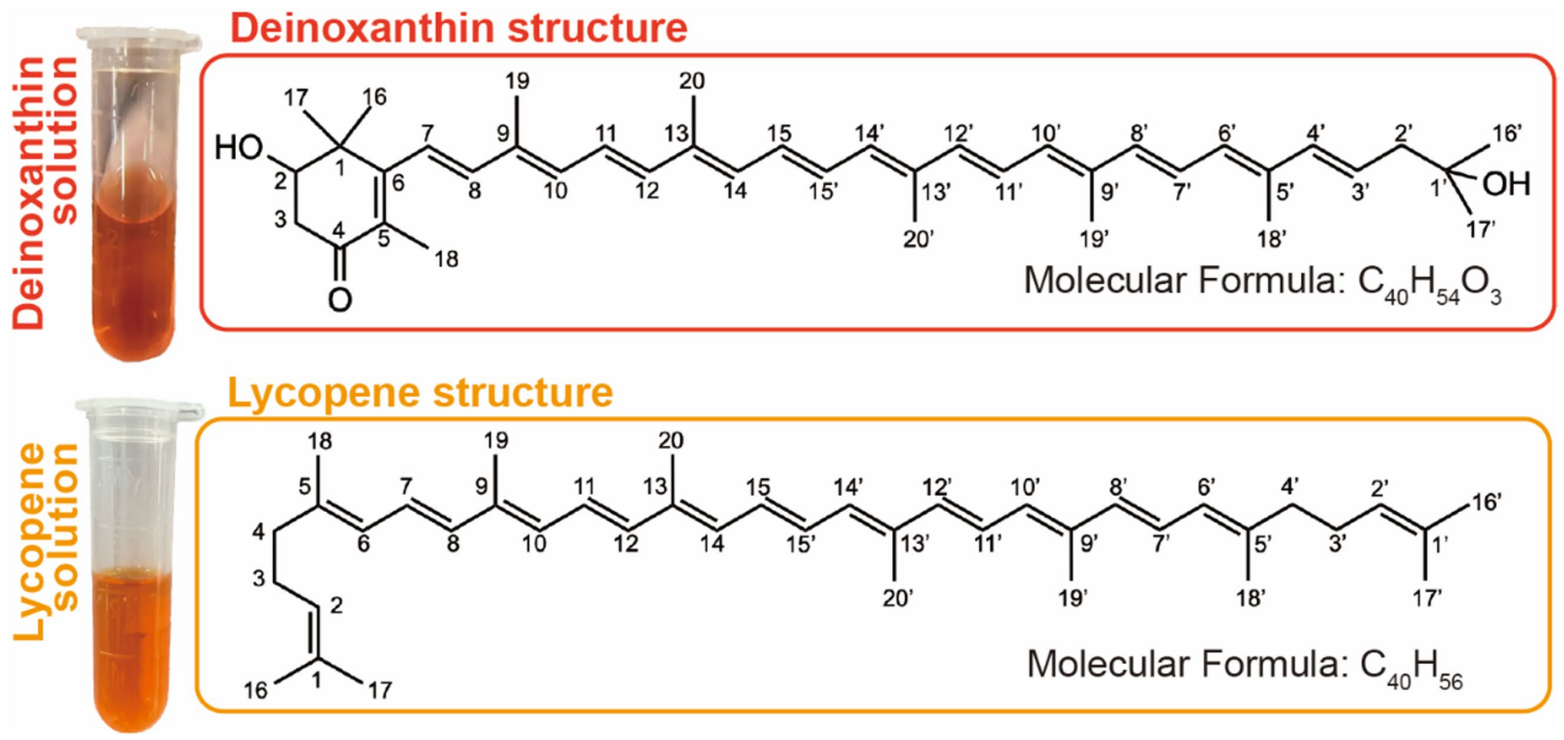
Deinoxanthin (up) and lycopene (down) solution and their chemical structure.

Deinoxanthin, a distinctive carotenoid, was first identified in *D. radiodurans*, which is a bacterium renowned for its remarkable resistance to ionizing radiation and other environmental stresses. The chemical structure of deinoxanthin is (all-E)-2,1′-dihydroxy-3′,4′-didehydro-1′,2′-dihydro-β, ψ-carotene-4-one-1 (Sadowska-Bartosz and Bartosz, 2023), which consists of an unsaturated carbon chain with a hydroxyl group on the end carbon atom (C-1′) and a hexatomic ring containing a hydroxyl group and a ketonic group on the C-2 and C-4 of the ring, respectively (Figure 1). The unique chemical structure forms the basis for its bio-function, as it contains 12 conjugated double bonds in the unsaturated carbon chain along with functional groups such as the hydroxyl group and ketonic group on the terminal ring (Figure 1). The antioxidant capacity of carotenoids is related to the length of their conjugated double bond system and the presence of functional groups (Albrecht et al., 2000). For example, bacterioruberin pigments with 13 conjugated double bonds exhibited superior antioxidant properties compared to β-carotene, which only comprises nine conjugated double bonds (Noby et al., 2023). Moreover, studies suggest that pigment color is also linked to the conjugated system, which can absorb specific wavelengths of light and thus produce its special color (Britton, 1995). It has been reported that conjugated systems with less than eight double bonds appear colorless to the human eyes and only absorb in the UV region. For example, there are 11 conjugated double bonds in the β-carotenoid and zeaxanthin with yellow color but none of them in the colorless phytoene, while the red color was observed in bacterioruberin due to the longer conjugated double bond system (Palanisamy and Ramalingam, 2024).

#### 3.1.2 Antioxidant effect of deinoxanthin

**Figure 2:**
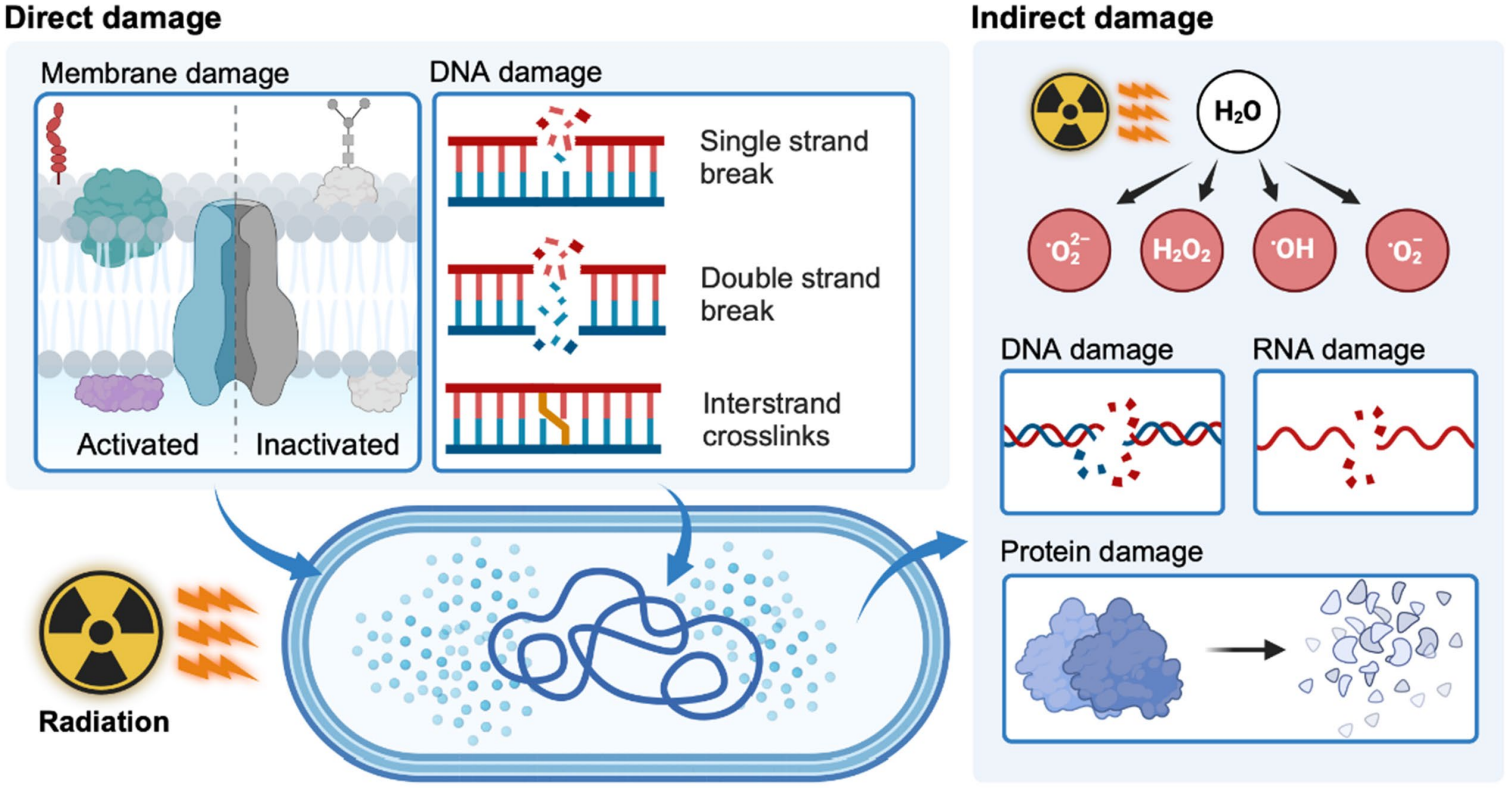
Mechanisms of direct and indirect radiation damage in bacteria. Created with BioRender.com.

The effects of ionizing radiation (e.g., γ-radiation and X-ray) on microbial cells are mainly caused by direct and indirect effects. The direct effect is that ionizing radiation can directly penetrate the cell membrane and destroy genetic material. The indirect effect is the radiolysis of water inside and outside the cells into reactive oxygen species (ROS), thereby indirectly damaging DNA, RNA, and protein (Figure 2) (Tian et al., 2004; Sadowska-Bartosz and Bartosz, 2023). The ROS-scavenging system is vital for *D. radiodurans* to decrease the impact of ROS damage, which consists of antioxidant enzymes (e.g., superoxide dismutase and catalase) and non-enzymatic antioxidants (e.g., carotenoids and metal ions). Carotenoids are strong ROS scavengers, especially singlet oxygen among non-enzymatic antioxidants (Tian et al., 2009). Deinoxanthin is a more effective ROS scavenger than other well-known carotenes, xanthophylls, and ascorbic acids (Cheng et al., 2014; Kim et al., 2021). It can react directly with free radicals and neutralize them (Yu et al., 2015). This effect is related to the conjugated double bond system in deinoxanthin that stabilizes unstable electrons formed by free radicals. Structural analysis of the S-layer deinoxanthin-binding complex in *D. radiodurans* further underscores the importance of deinoxanthin in the bacterium’s ability to withstand environmental challenges while highlighting its role in protective mechanisms against such challenges (Farci et al., 2022).

#### 3.1.3 Anticancer ability of deinoxanthin

Carotenoids such as β-carotene, lycopene, and lutein can induce apoptosis of a variety of cancer cells in animals and have an efficient effect in inhibiting carcinogenesis (Tanaka et al., 2012). The scavenging ability of deinoxanthin for active oxygen is stronger than that of these carotenoids. Therefore, it is also very important to study the anticancer effect of deinoxanthin. Current research has found that deinoxanthin can induce apoptosis in cancer cells such as liver cancer (Choi et al., 2014), colon cancer (Choi et al., 2014), prostate cancer (Choi et al., 2014), osteosarcoma (Tapia et al., 2021), and breast cancer (Tian et al., 2018; Günay and Kuzucu, 2023). Choi et al. (2014) found that deinoxanthin induced apoptosis through chromatin condensation and nuclear fragmentation in HepG2 liver cancer, HT-29 colon cancer, and PC-3 prostate cancer cells by releasing BAX and activating Caspase-3. Similarly, Tapia et al. (2021) found that deinoxanthin significantly reduced the proliferation activity of Saos-2 osteosarcoma cells by approximately 37.1%. In addition, tamoxifen has shown strong efficacy against breast cancer, and combining deinoxanthin with tamoxifen can enhance its anti-proliferation activity on cancer cells by leveraging its potential antioxidant effect and apoptosis-inducing ability (Günay and Kuzucu, 2023).

#### 3.1.4 Deinoxanthin derivatives

In addition to the well-known deinoxanthin, *D. radiodurans* also produces a unique carotenoid identified as (all-E)-1′-hydroxy-3′,4′-didehydro-1′,2′,2,3-quahydro-β, ψ-carotene-4-one-1 (Yang, 2021). The characterization of this carotenoid, named 2-deoxydeinoxanthin, is the precursor of deinoxanthin (Sadowska-Bartosz and Bartosz, 2023). The structural difference between this pigment and deinoxanthin is that the hydroxyl group on C-2 of the benzene ring is replaced by hydrogen. Despite lacking a hydroxyl group on C-2, it still contains many conjugated double bonds, suggesting its potential ability to resist ROS based on previous research. However, due to the absence of a hydroxyl group on C-2, its antioxidant capacity is theoretically weaker than that of deinoxanthin. Yang et al. found that 2-deoxydeinoxanthin could effectively scavenge radicals, including superoxide anion (O_2_^−^) and hydroxyl radical (·OH), thereby aiding in maintaining cell survival and recovery when exposed to radiation damage (Yang, 2021). Comparing the antioxidant and oxygen-free radical scavenging abilities of deinoxanthin and 2-deoxydeinoxanthin revealed that although the antioxidant capacity was weaker than that of deinoxanthin, 2-deoxydeinoxanthin still exhibited similar radiation protection effects (Yang, 2021). It plays a key role in *D. radiodurans*’ antioxidant defense system, particularly against oxidative stress caused by radiation exposure. These findings further emphasize the crucial role of carotenoids in *D. radiodurans*, especially in its extraordinary mechanism of radiation resistance.

### 3.2 Lycopene

#### 3.2.1 Physicochemical properties of lycopene

Lycopene belongs to the carotenoid family of compounds that are found in fruits, vegetables, and green plants. Like other carotenoids, lycopene is a natural pigment synthesized by plants and microorganisms that absorbs light during photosynthesis and protects them from photosensitization (Roldán-Gutiérrez and Castro, 2007). It is a non-cyclic carotenoid with a molecular formula of C_40_H_56_ and a molecular weight of 536.85 Da (Surmanidze et al., 2024). Being lipophilic, it appears as a red pigment that absorbs light in the visible range. The λ_max_ of lycopene in petroleum ether solution is 472 nm with ε^%^ of 3,450 (Rodríguez-Rodríguez et al., 2020). It consists of an 8-isoprene unit chain containing 40 carbons with 11 conjugated double bonds and two non-conjugated double bonds (Figure 1) (Wang, 2012). Similar to other carotenoids, their double bonds can be converted from trans to single or multiple cis isomers through exposure to light, thermal energy, or chemical reactions. However, due to the lack of β-ionone ring structure in the lycopene molecule, it does not have provitamin A activity (Ashraf et al., 2022).

#### 3.2.2 Lycopene and human health

Due to the special structure of lycopene, this pigment has strong antioxidant properties and anticancer activity, making it widely used in the food and medical industries (Khan et al., 2021). Studies have provided an overview of the mechanism of action of lycopene (Long et al., 2024). The main biological function of lycopene is its antioxidant properties. Research shows that consuming lycopene from the diet can increase its levels in circulation and tissues (Przybylska, 2020). As an antioxidant, it scavenges ROS and reduces oxidative stress and damage to cellular components including lipids, proteins, RNA, and DNA, therefore diminishing the risk of certain diseases such as cardiovascular disease, cancer, and osteoporosis by acting as a potent antioxidant (Caseiro et al., 2020; Kulawik et al., 2023). Numerous studies have demonstrated the association between lycopene intake and prevention/treatment of cancer. For example, daily consumption of lycopene may lower the risk of gut-related cancers as well as prostate cancer (Kapala et al., 2022). In addition, similar to deinoxanthin, lycopene exhibits a certain effect on breast cancer by inhibiting the proliferation of human MCF-7 breast cancer cells (Santos et al., 2018). Apart from its role in cancer treatment, lycopene also confers benefits for cardiovascular disease (Przybylska and Tokarczyk, 2022). Overall, lycopene has shown great potential in maintaining human health.

## 4 Deinoxanthin biosynthesis

### 4.1 Synthetic pathway

**Figure 3:**
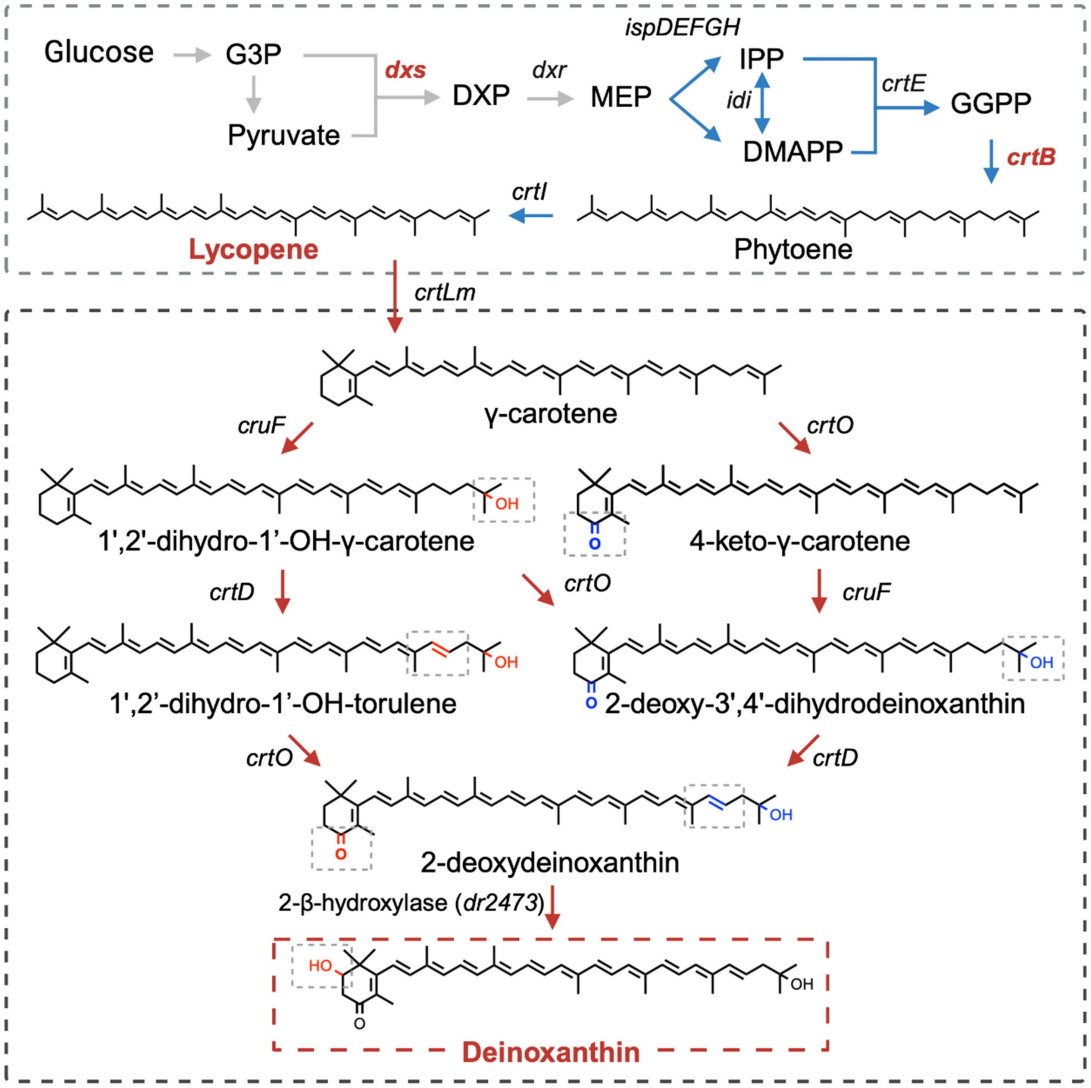
Deinoxanthin metabolic pathway (G3P: glyceraldehyde 3-phosphate; DXP: 1-deoxy-D-xylulose-5-phosphate; MEP: methylerythritol-4-phosphate; IPP: isopentenyl diphosphate; DMAPP: dimethylallyl diphosphate; GGPP: geranylgeranyl diphosphate; *dxs*: DXP synthase; *dxr*: DXP reductoisomerase; *ispD*: 4-(cytosine-5-phosphate)-2-C-methyl-D-erythritol (CDP-ME) synthase; *ispE*: CDP-ME kinase; *ispF*: 2-C-methyl-D-erythritol-2,4-diphosphate (MECP) synthase; *ispG*: 1-hydroxy-2-methyl-2-(E)-butenyl-4-diphosphate (HMBPP) synthase; *ispH*: HMBPP reductase; *idi*: isopentenyl diphosphate isomerase; *crtE*: GGPP synthase; *crtB*: phytoene synthase; *crtI*: phytoene desaturase; *crtLm*: lycopene cyclase; *cruF*: C-1′,2′-hydratase; *crtD*: C-3′,4′-desaturase; *crtO*: carotene ketolase). Created with BioRender.com.

Deinoxanthin is the main carotenoid product in the *Deinococcus* strains. Its synthesis begins with the general lycopene biosynthesis pathway, which involves multiple enzymes (Figure 3) (Du et al., 2023). Under the catalysis of various enzymes, the structure of lycopene becomes more complex, containing circular ends and multiple oxidative functional groups. First, under the catalysis of lycopene cyclase (CrtLm), the end of the lycopene molecule forms a β-ring to produce γ-carotene. Subsequently, through the catalysis of C-1′,2′-hydratase (CruF) and C-3′,4′-desaturase (CrtD), a double bond between C-1′ and C-2′ of γ-carotene is hydrated, and a hydroxyl group is added to C-1′, forming a double bond between C-3′ and C-4′. Additionally, carotene ketolase (CrtO) can introduce ketone groups at the C-4 position of γ-carotene and its derivatives. With these three enzymes acting together, γ-carotene is converted into 2-deoxydeinoxanthin, which serves as a precursor for deinoxanthin. Finally, 2-β-hydroxylase (DR2473) adds a hydroxyl group to the β-ring (C-2) of 2-deoxydeinoxanthin to generate deinoxanthin as its final product.

### 4.2 Synthetic enzymes

#### 4.2.1 Lycopene cyclase

The structure of some natural carotenoids is asymmetric and difficult to obtain by chemical synthesis (Su et al., 2023). However, there is a biological solution to solve this problem using enzymes. *Deinococcus* bacteria possess a type of lycopene cyclase (CrtLm), which is responsible for the cyclization of lycopene into various carotenoid products. CrtLm was identified in *D. radiodurans* by co-expressing *crtLm* with the *crtEIB* genes from *Pantoea stewartii* in *E. coli*. It is noteworthy that, unlike the CrtY-type lycopene β-cyclases that cyclize lycopene at both ends to produce β-lycopene, CrtLm selectively interacts with lycopene and asymmetrically cyclizes it into γ-carotene (Tao et al., 2004).

#### 4.2.2 C-1′,2′-hydratase

The end modification of monocyclic carotenoids requires carotenoid C-1′,2′-hydratase (CruF) to achieve C-1′,2′ hydration. A CruF type carotenoid-1′,2′-hydratase has been identified from *D. radiodurans* and *D. geothermalis*, which shows little homology with the CrtC type carotenoid hydratase mainly found in photosynthetic bacteria (Frigaard et al., 2004; Sun et al., 2009b). Phylogenetic analysis reveals that CruF homologs are clustered separately in a clade and have a distant evolutionary relationship with the CrtC homologs. The *cruF* gene in *Deinococcus* may have been obtained from a common ancestor or through horizontal gene transfer between bacteria (Sun et al., 2009b). The hydration process catalyzed by CruF is a key step in the carotenoid biosynthesis pathway, and the mechanism by which CruF converts carotenoids such as γ-carotene into hydroxyl compounds involves a crucial interaction between water and carbon–carbon double bond (Sun et al., 2009b).

#### 4.2.3 C-3′,4′-desaturase

The *crtD* gene in *D. radiodurans* (dr2250) is crucial for the desaturation process, specifically catalyzing the C-3′,4′-desaturation of the monocyclic precursor of deinoxanthin (Tian et al., 2008). This enzyme does not affect acyclic carotenoids. The C-3′,4′-desaturase (CrtD) from *D. radiodurans* specifically acts on carotenoids with a hydroxyl group at the C-1′ position. This specificity indicates that the enzyme preferentially modifies carotenoids that have already been modified by hydroxylation, rather than acting on initial carotenoid structure. Thus, the catalysis of CrtD during deinoxanthin synthesis always follows the reaction catalyzed by CruF. CrtD participates in the biosynthesis of deinoxanthin by catalyzing the desaturation at position C-3′,4′ on the carotenoid backbone. This action is critical in forming deinoxanthin, which contributes to the notable antioxidant properties and radiation resistance of *D. radiodurans* (Tian et al., 2008). Studies on similar enzymes in other bacteria have shown that CrtD-like enzymes could exhibit varied activities based on carotenoid structure. For instance, in *Rubrivivax gelatinosus*, a similar enzyme shows a preference for certain hydroxyspheroidene derivatives, highlighting the importance of specific structural features for enzyme activity (Steiger et al., 2000).

#### 4.2.4 Carotene ketolase

Carotene ketolase (CrtO) from *D. radiodurans* is capable of adding a keto group to the C-4 position of the β-ring of γ-carotene or its derivatives, producing ketoleted carotenoids. CrtO is part of the phytoene desaturase (CrtI) family, which includes carotene desaturases and carotenoid oxidases (Sun et al., 2009a). In *Synechocystis* sp., CrtO primarily acts as a mono-ketolase but can also produce small amounts of diketo canthaxanthin. The enzyme utilizes an oxidized quinone as a co-factor, and the catalytic process begins with a hydride transfer to the quinone, followed by a stabilization reaction forming a hydroxy group. The keto group is formed through two subsequent hydroxylations at the same C-atom and subsequent water elimination (Breitenbach et al., 2013). The mutation in CrtO led to a significant decrease in the ability of *D. radiodurans* R1 to convert γ-carotene derivatives into ketolated carotenoids. Moreover, this mutation resulted in crtOΔR1 exhibiting greater sensitivity to oxidative stress induced by hydrogen peroxide (H₂O₂) treatment compared to the wild-type strain. This increased sensitivity is attributed to the absence of the C-4 keto group on the carotenoids, which plays a critical role in their antioxidant activity. Additionally, carotenoid extracts from the mutant demonstrated lower radical scavenging activity, underscoring the importance of the C-4 keto group in the antioxidant defense mechanisms of *D. radiodurans* (Sun et al., 2009a). When expressed in *E. coli*, CrtO from *D. radiodurans* demonstrates its ability to convert accumulated β-carotene into ketocarotenoids, such as canthaxanthin. This indicates that functional expression of CrtO can be achieved in a heterologous host, potentially facilitating the production of valuable ketocarotenoids using a more controllable and scalable system (Tao and Cheng, 2004).

#### 4.2.5 2-β-hydroxylase

The 2-β-hydroxylase (DR2473) in *D. radiodurans* plays a crucial role in the synthesis of deinoxanthin, which is the final step in its biosynthesis pathway. 2-β-hydroxylase belongs to the class of cytochrome P450 monooxygenases that use electrons from NAD(P)H to activate molecular oxygen and perform specific oxidative attacks on structurally inactive carbon-hydrogen bonds of various chemicals (Werck-Reichhart and Feyereisen, 2000). In *D. radiodurans*, 2-β-hydroxylase adds a hydroxyl group to the β-ring (C-2) of 2-deoxydeinoxanthin to generate the final product of deinoxanthin (Zhou et al., 2015). Mutation of the gene *dr2473* encoding cytochrome P450 CYP287A1 leads to an accumulation of 2-deoxydeinoxanthin in *D. radiodurans*, suggesting that 2-β-hydroxylase can further convert it into other products. In addition, the Δ*dr2473* mutant exhibits increased sensitivity to UV radiation and oxidative stress compared to wild-type strain *D. radiodurans* R1, indicating that the hydroxyl group at the C-2 position is important for antioxidant activity (Zhou et al., 2015).

## 5 Pathway modification and heterologous expression

**Figure 4:**
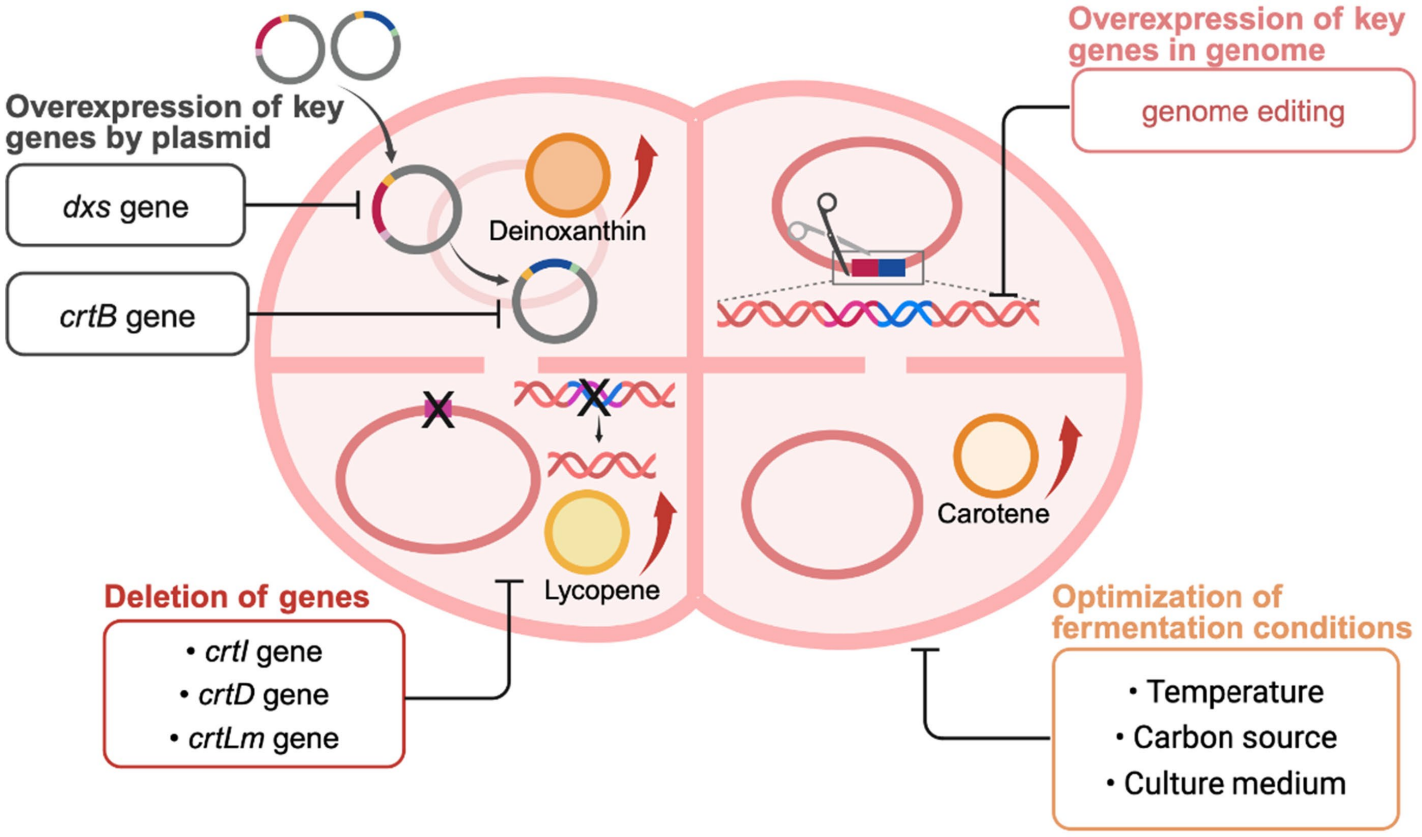
Metabolic engineering strategies for carotenoid biosynthesis in *Deinococcus*. Created with BioRender.com.

Metabolic engineering, an important technique in microbial bioengineering, has been widely used to enhance the production of specific metabolites, optimize biosynthetic pathways, and introduce new metabolic functions. *Deinococcus* has recently emerged as a promising target for microbial metabolic engineering due to its adaptability to extreme environments (Figure 4) (Tian et al., 2009). The production capacity of carotenoids in this genus is not only related to its remarkable radiation resistance but also attracts attention for potential applications in the pharmaceutical, food, and cosmetic industries (Cheng et al., 2014; Sadowska-Bartosz and Bartosz, 2023). However, the limited number of carotenoids naturally produced by these bacteria limits their industrial potential. Homologous expression of carotenoid products using *Deinococcus* as a chassis and heterologous expression of key exogenous genes in *Deinococcus* are widely utilized to enhance carotenoid production. Therefore, pathway modification (homologous expression) and heterologous expression based on metabolic engineering play crucial roles in improving carotenoid production.

### 5.1 Metabolic engineering of pigment synthesis in *Deinococcus*

#### 5.1.1 Overexpression of key genes

The overexpression of key genes is a direct way to increase the production of target metabolites. In *Deinococcus* bacteria, the activities of key enzymes in the carotenoid biosynthesis pathway, such as phytoene synthase (CrtB), directly affect the accumulation of end products. By increasing the expression of these enzymes through genetic engineering, it is possible to significantly increase the intracellular concentration of carotenoids. In carotenoid-producing strain, both the *dxs* gene encoding 1-deoxy-D-xylulose-5-phosphate synthase and *crtB* gene encoding phytoene synthase are considered rate-limiting steps in their respective carotenoid biosynthesis pathway (Rosas-Saavedra and Stange, 2016). However, genetic manipulation of the *Deinococcus* genus is difficult due to limitations in studying this genus, such as repetitive cloning steps and time-consuming processes. Additionally, the lack of tools for rescuing antibiotic resistance genes makes it impossible to create multiple knockout strains (Jeong et al., 2017). The application of Cre-lox-based rapid and efficient multiple knockout system in *D. radiodurans* R1 provides a new solution to this problem (Jeong et al., 2017). Based on this approach, the metabolic flux of deinoxanthin was enhanced by inserting *crtB* and *dxs* genes into the genome of *D. radiodurans* R1 (Jeong et al., 2021). The overexpression of *crtB* and *dxs* genes did not cause a burden on bacterial growth, and the deinoxanthin production of the engineered strain was 177.29 ± 8.4 mg/L, which was 200% higher than that of the wild-type strain. By comparing and analyzing mRNA expression levels for both genes, the optimal temperature was determined, and the carbon source was further optimized to significantly improve deinoxanthin production. The deinoxanthin production of the engineered strain was (394 ± 17.6) mg/L (102 ± 11.1 mg/g DCW) (Jeong et al., 2021). These studies on the overexpression of key genes provide a basis for the modification of metabolic pathways in *Deinococcus* bacteria. The overexpression of both *crtB* and *dxs* genes is necessary for subsequent studies on how to produce high amounts of carotenoids.

#### 5.1.2 Deletion of genes

The shunting of non-target carotenoids was reduced by knocking down specific *Crt*-like genes, thereby increasing the production of target carotenoids. For example, deleting the gene *crtI* that encodes phytoene desaturase cuts off the pathway for converting phytoene to downstream carotenoids, allowing for the accumulation of phytoene. The engineered strain using 10 g/L of fructose as a carbon source achieved a phytoene yield of 0.413 ± 0.023 mg/L (Jeong et al., 2018). Deleting the carotenoid 3′,4′-desaturase gene *crtD* and overexpressing *crtB* and *dxs* genes increased phytoene production in the engineered strain by 1,080% (4.46 ± 0.19 mg/L) (Jeong et al., 2017). Moreover, the *crtLm* gene encoding lycopene cyclase in *D. radiodurans* R1 was deleted to prevent the synthesis of γ-carotene, so that bacteria unable to produce lycopene could now produce it instead. Lycopene production was increased to 373.5 mg/L by constructing plasmids overexpressing *crtB* and *dxs* genes (Kang et al., 2020). Given the complexity of carotenoid metabolic pathways, one effective method for metabolic engineering to produce specific carotenoids is redirecting metabolic flow toward a specific pathway and deleting bypass genes.

#### 5.1.3 Optimization of fermentation conditions

The optimization of fermentation conditions is also a key factor in improving product yield. By finding the optimal growth conditions for microorganisms or providing abundant nutrients, the cell concentration can be increased to enhance the production of target products. To improve deinoxanthin production, cultivation conditions, especially temperature and carbon sources, have been optimized. The *groR* promoter in *D. radiodurans* has shown varying gene expression abilities at different temperatures (Schmid and Lidstrom, 2002). Therefore, regulation of *crtB* and *dxs* genes with the *groR* promoter was used to improve deinoxanthin production by optimizing fermentation temperature. At the optimal temperature (37°C), mRNA expression levels of *crtB* and *dxs* in the engineered strain were increased by 18 and 13 times, respectively, resulting in a deinoxanthin production of 256.5 ± 13.8 mg/L, which was 290% higher than that of the wild-type strain (Jeong et al., 2021). Additionally, replacing glucose with sucrose as the carbon source further enhanced deinoxanthin production in the engineered strain to reach 394 ± 17.6 mg/L, which was 446% higher than that of the wild-type strain (Jeong et al., 2021). Similarly, in *D.* sp. AJ005, the total carotenoid yield was increased by optimizing medium composition and sucrose concentrations. Specifically, a TGY medium containing 40 g/L of sucrose resulted in a total carotenoid yield that was 650% higher than that obtained without sucrose (Choi et al., 2019). As discussed in this review, the external conditions required for simulating extremophile habitats are an important consideration for their industrial production.

### 5.2 Heterologous expression of pigment synthesis genes in *Deinococcus*

In recent years, the use of genetic engineering techniques to exogenously express specific genes in model microorganisms for the production of target compounds has become a research hotspot in synthetic biology. As one of the model microorganisms, *E. coli* has been widely used in various biosynthetic studies due to its clear genetic background, easy genetic manipulation, and rapid growth (Adamczyk and Reed, 2017). To achieve efficient carotenoid production, the carotenoid synthesis genes from *Deinococcus* bacteria are introduced into *E. coli* and other model bacteria (Jin et al., 2015; Xu X. et al., 2018). Through molecular biological methods and bioengineering techniques, we can optimize the biosynthetic pathway of carotenoids and improve their expression and activity in host cells (Mori et al., 2022).

*Deinococcus wulumuqiensis* R12 has a strong antioxidant capacity and can produce deinoxanthin to improve radiation resistance (Wang et al., 2010; Xu et al., 2013). Three genes encoding carotenoid synthetase (*crtE*, *crtB*, and *crtI*) were identified from *D. wulumuqiensis* R12 to construct a polycistronic plasmid harboring these putative lycopene biosynthesis genes (*crtEBI*) for the production of lycopene. These genes were co-expressed in *E. coli* using the optimized Shine–Dalgarno (SD) region to increase lycopene yield and expressed in *E. coli* with different arrangements to study the effect of gene order. After optimizing the culture conditions, a high yield of lycopene was obtained, with the highest yield being 688 mg/L in the gene order *crtI-E-B* (Jin et al., 2015; Xu X. et al., 2018). However, an early study found that the gene order crtEBI (6.2 mg/g DCW) had the highest yield of lycopene, while *crtI-E-B* had the lowest yield (0.17 mg/g DCW) (Zhang et al., 2015). The reason for this opposite result may be attributed to differences in gene origin, as *crtI-E-B* had its highest yield from *D. wulumuqiensis* R12, while another study used three genes from three bacteria belonging to the genus *Pantoea*, respectively. In order to obtain *E. coli* with high lycopene production, experiments were conducted to find suitable plasmids for lycopene production and *E. coli* host strains (Xu J. et al., 2018). The lycopene synthesis genes *crtE*, *crtB*, and *crtI* from R12 were integrated into three different plasmids and then transferred into three different *E. coli* hosts, resulting in nine engineered strains. After the optimization of conditions, it was found that the selection of host and plasmid greatly impacted lycopene production, where medium copy number plasmids were more suitable than those with high or low copy numbers (Xu J. et al., 2018).

The production of deinoxanthin still needs to be industrialized at present, and research on heterologous expression of the deinoxanthin synthesis pathway has not been reported. Heterologous expression and metabolic engineering of deinoxanthin derived from *Deinococcus* will provide more experimental support for the further industrial production of deinoxanthin in more robust chassis cells.

## 6 Enhancement of bacterial resistance to environmental stress

Pigments play an important role in the survival and adaptation of bacteria, especially in response to external environmental stresses (Rao et al., 2017; Moors et al., 2021). In nature, many bacteria can produce different pigments, among which carotenoids are a class of pigment molecules that are ubiquitous and have diverse functions. Carotenoids not only give bacteria specific colors but also play key roles in antioxidant activity, photoprotection, and immune regulation (Chew and Park, 2004; Sipka and Maroti, 2018). By inhibiting ROS production and scavenging free radicals, carotenoids protect bacterial cells from damage caused by environmental stresses such as ultraviolet rays, oxidants, and high salt concentrations (Jones and Baxter, 2017). In recent years, with the progress of metabolic engineering technology, genetically modified strains capable of synthesizing carotenoids have been developed to significantly improve their tolerance to environmental stress (Albrecht et al., 2000). These engineered strains not only provide valuable models for basic research but also show broad prospects in industrial production and bioremediation applications (Albrecht et al., 2000). For example, introducing the carotenoid synthesis pathway into engineered bacteria exhibits enhanced viability and adaptability under conditions of high salt concentration, high temperature, and in the presence of oxidants. In addition, these modification techniques can be used to enhance the performance of industrial strains while improving the yield and stability of biological products.

Carotenoids in *Deinococcus* bacteria serve as non-enzymatic antioxidants, enabling them to resist oxidative toxicity in the external environment. *D. radiodurans* exhibited five orders of magnitude higher resistance to 10 mM H_2_O_2_ compared to *E. coli*, which does not produce deinoxanthin (Wang and Schellhorn, 1995). When *D. radiodurans* and its colorless mutant were exposed to different concentrations of H_2_O_2_, it was found that the wild-type strain showed no significant change in bacterial survival after treatment with various concentrations of H_2_O_2_ (0.83, 1.67, 4, and 5 mM), while the colorless mutant displayed a dose-dependent increase in sensitivity toward H_2_O_2_. At a concentration of 5 mM, it was found to be 100-fold more sensitive than the wild-type strain (Qi et al., 2020). In addition, genetic engineering techniques were used to knock out *crtB* and *crtI* genes involved in the deinoxanthin pathway of *D. radiodurans* R1, resulting in two colorless mutants that exhibited increased sensitivity to H_2_O_2_, ionizing radiation and UV (Zhang et al., 2007; Yang, 2021).

Further studies have found that the functional groups of deinoxanthin directly affect the antioxidant capacity of bacteria. When the gene for the last enzyme in the synthetic pathway (2-β-hydroxylase) is knocked out, the product is 2-deoxydeinoxanthin, with a hydroxyl group at C-2 of deinoxanthin. The LD_90_ of the mutant R1Δ*d2473* decreased from 71.2 mM/30 min in the wild type to 54.6 mM/30 min, indicating that deinoxanthin has a higher antioxidant capacity than 2-deoxydeinoxanthin (Zhou et al., 2015). The survival rate of the mutant R1Δ*d2473* was reduced by 90% at a UV irradiation dose of 1,000 J·m^−2^ compared to the wild type. The presence of a keto group at C-4 position in γ-carotene derivatives, formed by γ-carotene ketolase (CrtO), is crucial for this antioxidant activity (Sun et al., 2009a). Mutants lacking this keto group show increased sensitivity to oxidative stress and lower radical-scavenging activity, indicating the importance of structure of deinoxanthin in its antioxidant function as well. The product of R1Δ*crtD* mutant is 3′,4′-dihydrodeinoxanthin, which lacks a double bond between C-3′ and C-4′. The loss of this functional group significantly weakens the antioxidant capacity of 3′,4′-dihydrodeinoxanthin compared to deinoxanthin (*p* < 0.05) (Tian et al., 2008). Nevertheless, its antioxidant capacity is still higher than that of astaxanthin (Jeong et al., 2021).

## 7 Conclusion and prospects

This article reviews the synthesis and engineering of *Deinococcus* pigments, especially carotenoids, as well as their role in coping with environmental stress. First, we introduce the unique carotenoid synthesis pathway of *Deinococcus* species, including the functions and mechanisms of key enzymes. Next, strategies for improving carotenoid production through metabolic engineering are discussed, such as overexpression of key genes, gene knockout, and optimization of fermentation conditions. Finally, we explore the key role of these carotenoids in bacterial resistance to oxidative stress and use genetic modification and heterologous expression techniques to enable other bacteria to synthesize these carotenoids and enhance their stress resistance. In conclusion, carotenoids in *Deinococcus* not only play important roles in their survival and environmental adaptability but also have potential industrial applications. An in-depth study of these bacterial pigments will not only reveal their unique biological mechanisms but also provide new ideas and methods for biotechnology and industrial applications. Future research in pigment production by *Deinococcus* bacteria can be carried out from the following aspects:

1. Gene regulation and metabolic pathway optimization. Future studies should further reveal the gene regulation mechanism of carotenoid synthesis in *Deinococcus* species and optimize metabolic pathways to improve pigment production. For example, gene editing technologies such as CRISPR/Cas9 can be used to precisely regulate the expression of key enzyme genes and increase the amount of carotenoid accumulation. Additionally, metabolic flux analysis can be used to identify and eliminate metabolic bottlenecks for further improvement in pigment production.
2. Synthetic biology and heterologous expression. The development of synthetic biology provides a new idea for the heterologous synthesis of carotenoids. The carotenoid synthesis gene from *Deinococcus* is cloned into industrial microorganisms such as *E. coli*, and efficient production is achieved by optimizing the expression vector and fermentation conditions. In addition, new pigments and derivatives are developed using modular design and combinatorial biosynthetic pathways.
3. Application in space exploration. Carotenoids from *Deinococcus* bacteria are highly radioresistant, and previous studies have shown their excellent performance in space exploration, which holds significant potential for practical applications (Ott et al., 2019, 2020). For example, these pigments could serve as radiation indicators to monitor real-time levels of radiation exposure for astronauts and equipment. In addition, these pigments can also act as natural antioxidants to protect the health of astronauts during long-term spaceflight.
4. Biotechnology and pharmaceutical applications. Carotenoids from *Deinococcus* bacteria have broad prospects for applications in food, pharmaceutical, and cosmetic fields. For example, the antioxidant and anticancer properties of deinoxanthin can be exploited to develop new functional foods and nutritional supplements. At the same time, these pigments can also be used as natural substitutes for synthetic pigments to meet the market demand for natural products.

## Author contributions

YW: Conceptualization, Funding acquisition, Methodology, Validation, Writing – original draft. JL: Conceptualization, Methodology, Validation, Writing – original draft. YY: Methodology, Writing – original draft. LZ: Formal analysis, Investigation, Writing – original draft. ML: Formal analysis, Investigation, Writing – review & editing. ZZ: Funding acquisition, Writing – review & editing. QX: Validation, Writing – review & editing. LJ: Funding acquisition, Writing – review & editing.

## References

[ref1] Adamczyk P. A. Reed J. L. (2017). *Escherichia coli* as a model organism for systems metabolic engineering. Curr. Opin. Syst. Biol. 6, 80–88. doi: 10.1016/j.coisb.2017.11.001

[ref2] Albrecht M. Takaichi S. Steiger S. Wang Z. Sandmann G. (2000). Novel hydroxycarotenoids with improved antioxidative properties produced by gene combination in *Escherichia coli*. Nat. Biotechnol. 18, 843–846. doi: 10.1038/78443, PMID: 10932152

[ref3] Ashraf W. Latif A. Zhang T. Zhang J. Wang C. Rehman A.. (2022). Technological advancement in the processing of lycopene: a review. Food Rev. Int. 38, 857–883. doi: 10.1080/87559129.2020.1749653

[ref4] Basu B. (2022). The radiophiles of *Deinococcaceae* family: resourceful microbes for innovative biotechnological applications. Curr. Res. Microb. Sci. 3:100153. doi: 10.1016/j.crmicr.2022.100153, PMID: 35909625 PMC9325910

[ref5] Breitenbach J. Gerjets T. Sandmann G. (2013). Catalytic properties and reaction mechanism of the CrtO carotenoid ketolase from the cyanobacterium *Synechocystis sp.* PCC 6803. Arch. Biochem. Biophys. 529, 86–91. doi: 10.1016/j.abb.2012.11.003, PMID: 23220023

[ref6] Britton G. (1995). Structure and properties of carotenoids in relation to function. FASEB J. 9, 1551–1558. doi: 10.1096/fasebj.9.15.8529834 8529834

[ref7] Caseiro M. Ascenso A. Costa A. Creagh-Flynn J. Johnson M. Simoes S. (2020). Lycopene in human health. LWT 127:109323. doi: 10.1016/j.lwt.2020.109323

[ref8] Celedón R. S. Díaz L. B. (2021). Natural pigments of bacterial origin and their possible biomedical applications. Microorganisms 9:739. doi: 10.3390/microorganisms9040739, PMID: 33916299 PMC8066239

[ref9] Chen A. Hernandez-Vargas J. Han R. Cortazar-Martinez O. Gonzalez N. Patel S.. (2021). Small RNAs as a new platform for tuning the biosynthesis of silver nanoparticles for enhanced material and functional properties. ACS Appl. Mater. Interfaces 13, 36769–36783. doi: 10.1021/acsami.1c07400, PMID: 34319072

[ref10] Cheng J. Zhang Z. Zheng Z. Lv G. Wang L. Tian B.. (2014). Antioxidative and hepatoprotective activities of deinoxanthin-rich extract from *Deinococcus radiodurans* R1 against carbon tetrachloride-induced liver injury in mice. Trop. J. Pharm. Res. 13, 581–586. doi: 10.4314/tjpr.v13i4.14

[ref11] Chew B. P. Park J. S. (2004). Carotenoid action on the immune response. J. Nutr. 134, 257S–261S. doi: 10.1093/jn/134.1.257S 14704330

[ref12] Choi Y. J. Hur J. M. Lim S. Jo M. Kim D. H. Choi J. I. (2014). Induction of apoptosis by deinoxanthin in human cancer cells. Anticancer Res. 34, 1829–1835 Available at: https://ar.iiarjournals.org/content/34/4/1829., PMID: 24692716

[ref13] Choi J. Y. Lee K. Lee P. C. (2019). Characterization of carotenoid biosynthesis in newly isolated *Deinococcus* sp. AJ005 and investigation of the effects of environmental conditions on cell growth and carotenoid biosynthesis. Mar. Drugs 17:705. doi: 10.3390/md17120705, PMID: 31847382 PMC6950390

[ref14] Daly M. J. (2012). Death by protein damage in irradiated cells. DNA Repair 11, 12–21. doi: 10.1016/j.dnarep.2011.10.024 22112864

[ref15] Du B. Sun M. Hui W. Xie C. Xu X. (2023). Recent advances on key enzymes of microbial origin in the lycopene biosynthesis pathway. J. Agric. Food Chem. 71, 12927–12942. doi: 10.1021/acs.jafc.3c03942, PMID: 37609695

[ref16] Esteban R. Moran J. F. Becerril J. M. García-Plazaola J. I. (2015). Versatility of carotenoids: an integrated view on diversity, evolution, functional roles and environmental interactions. Environ. Exp. Bot. 119, 63–75. doi: 10.1016/j.envexpbot.2015.04.009

[ref17] Farci D. Haniewicz P. Sanctis D. Lesu L. Kereiche S. Winterhalter M.. (2022). The cryo-EM structure of the S-layer deinoxanthin-binding complex of *Deinococcus radiodurans* informs properties of its environmental interactions. J. Biol. Chem. 298:102031. doi: 10.1016/j.jbc.2022.102031, PMID: 35577074 PMC9189128

[ref18] Flieger J. Raszewska-Famielec M. Radzikowska-Büchner E. Flieger W. (2024). Skin protection by carotenoid pigments. Int. J. Mol. Sci. 25:1431. doi: 10.3390/ijms25031431, PMID: 38338710 PMC10855854

[ref19] Frigaard N. U. Maresca J. A. Yunker C. E. Jones A. D. Bryant D. A. (2004). Genetic manipulation of carotenoid biosynthesis in the green sulfur bacterium *Chlorobium tepidum*. J. Bacteriol. 186, 5210–5220. doi: 10.1128/jb.186.16.5210-5220.2004, PMID: 15292122 PMC490927

[ref20] Ghosh S. Sarkar T. Chakraborty R. Shariati M. A. Simal-Gandara J. (2024). Nature’s palette: an emerging frontier for coloring dairy products. Crit. Rev. Food Sci. Nutr. 64, 1508–1552. doi: 10.1080/10408398.2022.2117785, PMID: 36066466

[ref21] Giani M. Pire C. Martinez-Espinosa R. (2024). Bacterioruberin: biosynthesis, antioxidant activity, and therapeutic applications in cancer and immune pathologies. Mar. Drugs 22:167. doi: 10.3390/md22040167, PMID: 38667784 PMC11051356

[ref22] Griffiths E. Gupta R. S. (2004). Distinctive protein signatures provide molecular markers and evidence for the monophyletic nature of the *Deinococcus-Thermus* phylum. J. Bacteriol. 186, 3097–3107. doi: 10.1128/jb.186.10.3097-3107.2004, PMID: 15126471 PMC400596

[ref23] Günay N. Kuzucu M. (2023). Agonistic effects of deinoxanthin on tamoxifen antiproliferative activity on HER2 positive breast cancer: an *in vitro* study on MDA-MB-453. Erzincan Univ. J. Sci. Technol. 16, 138–154. doi: 10.18185/erzifbed.1224499

[ref24] Han R. Fang J. Jiang J. Gaidamakova E. K. Tkava R. Daly M. J.. (2020). Signal recognition particle RNA contributes to oxidative stress response in *Deinococcus radiodurans* by modulating catalase localization. Front. Microbiol. 11:613571. doi: 10.3389/fmicb.2020.613571, PMID: 33391243 PMC7775534

[ref25] Han R. Jiang J. Fang J. Contreras L. M. (2022). PNPase and RhlB interact and reduce the cellular availability of oxidized RNA in *Deinococcus radiodurans*. Microbiol. Spectr. 10, e02140–e02122. doi: 10.1128/spectrum.02140-22, PMID: 35856907 PMC9430589

[ref26] Jeong S. W. Kang C. K. Choi Y. J. (2018). Metabolic engineering of *Deinococcus radiodurans* for the production of phytoene. J. Microbiol. Biotechnol. 28, 1691–1699. doi: 10.4014/jmb.1808.08019, PMID: 30178642

[ref27] Jeong S. W. Kim J. H. Kim J. W. Kim C. Y. Kim S. Y. Choi Y. J. (2021). Metabolic engineering of extremophilic bacterium *Deinococcus radiodurans* for the production of the novel carotenoid deinoxanthin. Microorganisms 9:44. doi: 10.3390/microorganisms9010044, PMID: 33375757 PMC7823818

[ref28] Jeong S. W. Yang J. E. Im S. Choi Y. J. (2017). Development of Cre-*lox* based multiple knockout system in *Deinococcus radiodurans* R1. Korean J. Chem. Eng. 34, 1728–1733. doi: 10.1007/s11814-017-0082-5

[ref29] Jin W. Xu X. Jiang L. Zhang Z. Li C. Huang H. (2015). Putative carotenoid genes expressed under the regulation of Shine-Dalgarno regions in *Escherichia coli* for efficient lycopene production. Biotechnol. Lett. 37, 2303–2310. doi: 10.1007/s10529-015-1922-1, PMID: 26253301

[ref30] Jones D. L. Baxter B. K. (2017). DNA repair and photoprotection: mechanisms of overcoming environmental ultraviolet radiation exposure in halophilic archaea. Front. Microbiol. 8:1882. doi: 10.3389/fmicb.2017.01882, PMID: 29033920 PMC5626843

[ref31] Kang C. K. Jeong S. W. Yang J. E. Choi Y. J. (2020). High-yield production of lycopene from corn steep liquor and glycerol using the metabolically engineered *Deinococcus radiodurans* R1 strain. J. Agric. Food Chem. 68, 5147–5153. doi: 10.1021/acs.jafc.0c01024, PMID: 32275417

[ref32] Kapala A. Szlendak M. Motacka E. (2022). The anti-cancer activity of lycopene: a systematic review of human and animal studies. Nutrients 14:5152. doi: 10.3390/nu14235152, PMID: 36501182 PMC9741066

[ref33] Khan U. M. Sevindik M. Zarrabi A. Nami M. Ozdemir B. Kaplan D. N.. (2021). Lycopene: food sources, biological activities, and human health benefits. Oxidative Med. Cell. Longev. 2021:2713511. doi: 10.1155/2021/2713511, PMID: 34840666 PMC8626194

[ref34] Kim W. Kim M. Hong M. Park W. (2021). Killing effect of deinoxanthins on cyanobloom-forming *Microcystis aeruginosa*: eco-friendly production and specific activity of deinoxanthins. Environ. Res. 200:111455. doi: 10.1016/j.envres.2021.111455, PMID: 34118245

[ref35] Kulawik A. Cielecka-Piontek J. Zalewski P. (2023). The importance of antioxidant activity for the health-promoting effect of lycopene. Nutrients 15:3821. doi: 10.3390/nu15173821, PMID: 37686853 PMC10490373

[ref36] Liang M. Zhu J. Jiang J. (2018). Carotenoids biosynthesis and cleavage related genes from bacteria to plants. Crit. Rev. Food Sci. Nutr. 58, 2314–2333. doi: 10.1080/10408398.2017.1322552, PMID: 28609133

[ref37] Lim S. Jung J. H. Blanchard L. Groot A. D. (2019). Conservation and diversity of radiation and oxidative stress resistance mechanisms in *Deinococcus* species. FEMS Microbiol. Rev. 43, 19–52. doi: 10.1093/femsre/fuy037, PMID: 30339218 PMC6300522

[ref38] Long Y. Paengkoum S. Lu S. Niu X. Thongpea S. Taethaisong N.. (2024). Physicochemical properties, mechanism of action of lycopene and its application in poultry and ruminant production. Front. Vet. Sci. 11:1364589. doi: 10.3389/fvets.2024.1364589, PMID: 38562916 PMC10983797

[ref39] Luan H. Meng N. Fu J. Chen X. Xu X. Feng Q.. (2014). Genome-wide transcriptome and antioxidant analyses on gamma-irradiated phases of *Deinococcus radiodurans* R1. PLoS One 9:e85649. doi: 10.1371/journal.pone.0085649, PMID: 24465634 PMC3900439

[ref40] Maoka T. (2020). Carotenoids as natural functional pigments. J. Nat. Med. 74, 1–16. doi: 10.1007/s11418-019-01364-x, PMID: 31588965 PMC6949322

[ref41] Moors K. A. Ott E. Weckwerth W. Milojevic T. (2021). Proteomic response of *Deinococcus radiodurans* to short-term real microgravity during parabolic flight reveals altered abundance of proteins involved in stress response and cell envelope functions. Life 12:23. doi: 10.3390/life12010023, PMID: 35054415 PMC8779699

[ref42] Mori H. Kataoka M. Yang X. (2022). Past, present, and future of genome modification in *Escherichia coli*. Microorganisms 10:1835. doi: 10.3390/microorganisms10091835, PMID: 36144436 PMC9504249

[ref43] Nayak T. Sengupta I. Dhal P. K. (2021). A new era of radiation resistance bacteria in bioremediation and production of bioactive compounds with therapeutic potential and other aspects: an in-perspective review. J. Environ. Radioact. 237:106696. doi: 10.1016/j.jenvrad.2021.106696, PMID: 34265519

[ref44] Noby N. Khattab S. N. Soliman N. A. (2023). Sustainable production of bacterioruberin carotenoid and its derivatives from *Arthrobacter agilis* NP20 on whey-based medium: optimization and product characterization. Bioresour. Bioprocess. 10:46. doi: 10.1186/s40643-023-00662-3, PMID: 38647623 PMC10991996

[ref45] Orlandi V. T. Martegani E. Giaroni C. Baj A. Bolognese F. (2022). Bacterial pigments: a colorful palette reservoir for biotechnological applications. Biotechnol. Appl. Biochem. 69, 981–1001. doi: 10.1002/bab.2170, PMID: 33870552 PMC9544673

[ref46] Ott E. Fuchs F. M. Moeller R. Hemmersbach R. Kawaguchi Y. Yamagishi A.. (2019). Molecular response of *Deinococcus radiodurans* to simulated microgravity explored by proteometabolomic approach. Sci. Rep. 9:18462. doi: 10.1038/s41598-019-54742-6, PMID: 31804539 PMC6895123

[ref47] Ott E. Kawaguchi Y. Kolbl D. Rabbow E. Rettberg P. Mora M.. (2020). Molecular repertoire of *Deinococcus radiodurans* after 1 year of exposure outside the international Space Station within the Tanpopo mission. Microbiome 8:150. doi: 10.1186/s40168-020-00927-5, PMID: 33121542 PMC7597052

[ref48] Palanisamy M. Ramalingam S. (2024). Microbial bacterioruberin: A comprehensive review. Indian J. Microbiol., 1–25. In Press. doi: 10.1007/s12088-024-01312-838468730

[ref49] Park S. A. Ahn S. Y. Choi K. Y. (2020). Functional microbial pigments isolated from *Chryseobacterium* and *Deinococcus* species for bio-paint application. Biotechnol. Bioprocess Eng. 25, 394–402. doi: 10.1007/s12257-019-0372-3

[ref50] Poli A. Finore I. Romano I. Gioiello A. Lama L. Nicolaus B. (2017). Microbial diversity in extreme marine habitats and their biomolecules. Microorganisms 5:25. doi: 10.3390/microorganisms5020025, PMID: 28509857 PMC5488096

[ref51] Przybylska S. (2020). Lycopene–a bioactive carotenoid offering multiple health benefits: a review. Int. J. Food Sci. Technol. 55, 11–32. doi: 10.1111/ijfs.14260

[ref52] Przybylska S. Tokarczyk G. (2022). Lycopene in the prevention of cardiovascular diseases. Int. J. Mol. Sci. 23:1957. doi: 10.3390/ijms23041957, PMID: 35216071 PMC8880080

[ref53] Qi H. Wang W. He J. Ma Y. Xiao F. He S. (2020). Antioxidative system of *Deinococcus radiodurans*. Res. Microbiol. 171, 45–54. doi: 10.1016/j.resmic.2019.11.002 31756434

[ref54] Rajendran P. Somasundaram P. Dufossé L. (2023). Microbial pigments: eco-friendly extraction techniques and some industrial applications. J. Mol. Struct. 1290:135958. doi: 10.1016/j.molstruc.2023.135958

[ref55] Rao M. P. N. Xiao M. Li W. J. (2017). Fungal and bacterial pigments: secondary metabolites with wide applications. Front. Microbiol. 8:1113. doi: 10.3389/fmicb.2017.01113, PMID: 28690593 PMC5479939

[ref56] Rodríguez-Rodríguez E. Beltrán-de-Miguel B. Samaniego-Aguilar K. X. Sánchez-Prieto M. Estévez-Santiago R. Olmedilla-Alonso B. (2020). Extraction and analysis by HPLC-DAD of carotenoids in human faeces from Spanish adults. Antioxidants 9:484. doi: 10.3390/antiox9060484, PMID: 32503206 PMC7346146

[ref57] Roldán-Gutiérrez J. M. Castro M. D. L. (2007). Lycopene: the need for better methods for characterization and determination. *TrAC*. Trends Anal. Chem. 26, 163–170. doi: 10.1016/j.trac.2006.11.013

[ref58] Romanovskaya V. Rokitko P. Mikheev A. Gushcha N. I. Malashenko Y. R. Chernaya N. A. (2002). The effect of γ-radiation and desiccation on the viability of the soil bacteria isolated from the alienated zone around the Chernobyl nuclear power plant. Microbiology 71, 608–613. doi: 10.1023/A:1020575223365 12449639

[ref59] Rosas-Saavedra C. Stange C. (2016). Biosynthesis of carotenoids in plants: enzymes and color. Subcell. Biochem. 79, 35–69. doi: 10.1007/978-3-319-39126-7_2 27485218

[ref60] Sadowska-Bartosz I. Bartosz G. (2023). Antioxidant defense of *Deinococcus radiodurans*: how does it contribute to extreme radiation resistance? Int. J. Radiat. Biol. 99, 1803–1829. doi: 10.1080/09553002.2023.2241895, PMID: 37498212

[ref61] Santos R. C. Ombredane A. S. Souza J. M. T. Vasconcelos A. G. Placido A. Amorim A. G. N.. (2018). Lycopene-rich extract from red guava (*Psidium guajava* L.) displays cytotoxic effect against human breast adenocarcinoma cell line MCF-7 via an apoptotic-like pathway. Food Res. Int. 105, 184–196. doi: 10.1016/j.foodres.2017.10.045, PMID: 29433206

[ref62] Schmid A. K. Lidstrom M. E. (2002). Involvement of two putative alternative sigma factors in stress response of the radioresistant bacterium *Deinococcus radiodurans*. J. Bacteriol. 184, 6182–6189. doi: 10.1128/jb.184.22.6182-6189.2002, PMID: 12399488 PMC151957

[ref63] Sen T. Barrow C. J. Deshmukh S. K. (2019). Microbial pigments in the food industry-challenges and the way forward. Front. Nutr. 6:7. doi: 10.3389/fnut.2019.00007, PMID: 30891448 PMC6411662

[ref64] Shu W. S. Huang L. N. (2022). Microbial diversity in extreme environments. Nat. Rev. Microbiol. 20, 219–235. doi: 10.1038/s41579-021-00648-y 34754082

[ref65] Sipka G. Maroti P. (2018). Photoprotection in intact cells of photosynthetic bacteria: quenching of bacteriochlorophyll fluorescence by carotenoid triplets. Photosynth. Res. 136, 17–30. doi: 10.1007/s11120-017-0434-3, PMID: 29064080

[ref66] Steiger S. Astier C. Sandmann G. (2000). Substrate specificity of the expressed carotenoid 3,4-desaturase from *Rubrivivax gelatinosus* reveals the detailed reaction sequence to spheroidene and spirilloxanthin. Biochem. J. 349, 635–640. doi: 10.1042/bj3490635 10880364 PMC1221188

[ref67] Su B. Deng M. R. Zhu H. (2023). Advances in the discovery and engineering of gene targets for carotenoid biosynthesis in recombinant strains. Biomol. Ther. 13:1747. doi: 10.3390/biom13121747, PMID: 38136618 PMC10742120

[ref68] Sun Z. Shen S. Tian B. Wang H. Xu Z. Wang L.. (2009a). Functional analysis of γ-carotene ketolase involved in the carotenoid biosynthesis of *Deinococcus radiodurans*. FEMS Microbiol. Lett. 301, 21–27. doi: 10.1111/j.1574-6968.2009.01794.x, PMID: 19832905

[ref69] Sun Z. Shen S. Wang C. Wang H. Hu Y. Jiao J.. (2009b). A novel carotenoid 1,2-hydratase (CruF) from two species of the non-photosynthetic bacterium *Deinococcus*. Microbiology 155, 2775–2783. doi: 10.1099/mic.0.027623-0, PMID: 19443548

[ref70] Sun H. Xu G. Zhan H. Chen H. Sun Z. Tian B.. (2010). Identification and evaluation of the role of the manganese efflux protein in *Deinococcus radiodurans*. BMC Microbiol. 10, 1–8. doi: 10.1186/1471-2180-10-319, PMID: 21156049 PMC3016326

[ref71] Surmanidze N. Vanidze M. Djafaridze I. Davitadze R. Qarcivadze I. Khakhutaishvili M.. (2024). Optimization of the method of ultrasonic extraction of lycopene with a green extract from the fruit of *Elaeagnus umbellata*, common in Western Georgia. Food Sci. Nutr. 12, 3593–3601. doi: 10.1002/fsn3.4030, PMID: 38726431 PMC11077213

[ref72] Tanaka T. Shnimizu M. Moriwaki H. (2012). Cancer chemoprevention by carotenoids. Molecules 17, 3202–3242. doi: 10.3390/molecules17033202, PMID: 22418926 PMC6268471

[ref73] Tao L. Cheng Q. (2004). Novel beta-carotene ketolases from non-photosynthetic bacteria for canthaxanthin synthesis. Mol. Gen. Genomics. 272, 530–537. doi: 10.1007/s00438-004-1083-8, PMID: 15538629

[ref74] Tao L. Picataggio S. Rouviere P. E. Cheng Q. (2004). Asymmetrically acting lycopene beta-cyclases (CrtLm) from non-photosynthetic bacteri. Mol. Gen. Genomics. 271, 180–188. doi: 10.1007/s00438-003-0969-1, PMID: 14740205

[ref75] Tapia C. Lopez B. Astuya A. Becerra J. Gugliandolo C. Parra B. (2021). Antiproliferative activity of carotenoid pigments produced by extremophile bacteria. Nat. Prod. Res. 35, 4638–4642. doi: 10.1080/14786419.2019.1698574, PMID: 31809588

[ref76] Tian B. Hua Y. (2010). Carotenoid biosynthesis in extremophilic *Deinococcus–Thermus* bacteria. Trends Microbiol. 18, 512–520. doi: 10.1016/j.tim.2010.07.007 20832321

[ref77] Tian B. Li J. Pang R. Dai S. Li T. Weng Y.. (2018). Gold nanoparticles biosynthesized and functionalized using a hydroxylated tetraterpenoid trigger gene expression changes and apoptosis in cancer cells. ACS Appl. Mater. Interfaces 10, 37353–37363. doi: 10.1021/acsami.8b09206, PMID: 30295457

[ref78] Tian B. Sun Z. Shen S. Wang H. Jiao J. Wang L.. (2009). Effects of carotenoids from *Deinococcus radiodurans* on protein oxidation. Lett. Appl. Microbiol. 49, 689–694. doi: 10.1111/j.1472-765X.2009.02727.x 19780959

[ref79] Tian B. Sun Z. Xu Z. Shen S. Wang H. Hua Y. (2008). Carotenoid 3′, 4′-desaturase is involved in carotenoid biosynthesis in the radioresistant bacterium *Deinococcus radiodurans*. Microbiology 154, 3697–3706. doi: 10.1099/mic.0.2008/021071-0 19047737

[ref80] Tian B. Wu Y. Sheng D. Zheng Z. Gao G. Hua Y. (2004). Chemiluminescence assay for reactive oxygen species scavenging activities and inhibition on oxidative damage of DNA in *Deinococcus radiodurans*. Luminescence 19, 78–84. doi: 10.1002/bio.761, PMID: 15098207

[ref81] Venil C. K. Zakaria Z. A. Ahmad W. A. (2013). Bacterial pigments and their applications. Process Biochem. 48, 1065–1079. doi: 10.1016/j.procbio.2013.06.006

[ref82] Villa J. K. Han R. Tsai C. H. Chen A. Sweet P. Franco G.. (2021). A small RNA regulates *pprM*, a modulator of pleiotropic proteins promoting DNA repair, in *Deinococcus radiodurans* under ionizing radiation. Sci. Rep. 11:12949. doi: 10.1038/s41598-021-91335-8, PMID: 34155239 PMC8217566

[ref83] Wang X. (2012). Lycopene metabolism and its biological significance. Am. J. Clin. Nutr. 96, 1214S–1222S. doi: 10.3945/ajcn.111.032359, PMID: 23053559 PMC3471203

[ref84] Wang W. Mao J. Zhang Z. Tang Q. Xie Y. Zhu J.. (2010). *Deinococcus wulumuqiensis* sp. nov., and *Deinococcus xibeiensis* sp. nov., isolated from radiation-polluted soil. Int. J. Syst. Evol. Microbiol. 60, 2006–2010. doi: 10.1099/ijs.0.015917-0, PMID: 19801390

[ref85] Wang P. Schellhorn H. E. (1995). Induction of resistance to hydrogen peroxide and radiation in *Deinococcus radiodurans*. Can. J. Microbiol. 41, 170–176. doi: 10.1139/m95-023, PMID: 7720013

[ref86] Werck-Reichhart D. Feyereisen R. (2000). Cytochromes P450: a success story. Genome Biol. 1:REVIEWS3003. doi: 10.1186/gb-2000-1-6-reviews3003, PMID: 11178272 PMC138896

[ref87] Wurtzel E. T. (2019). Changing form and function through carotenoids and synthetic biology. Plant Physiol. 179, 830–843. doi: 10.1104/pp.18.01122, PMID: 30361256 PMC6393808

[ref88] Xu X. Jiang L. Zhang Z. Shi Y. Huang H. (2013). Genome sequence of a gamma-and UV-ray-resistant strain, *Deinococcus wulumuqiensis* R12[J]. Genome Announc. 1:e00206. doi: 10.1128/genomea.00206-13 23661483 PMC3650442

[ref89] Xu X. Tian L. Xu J. Xie C. Jiang L. Huang H. (2018). Analysis and expression of the carotenoid biosynthesis genes from *Deinococcus wulumuqiensis* R12 in engineered *Escherichia coli*. AMB Expr. 8:94. doi: 10.1186/s13568-018-0624-1, PMID: 29860613 PMC5984946

[ref90] Xu J. Xu X. Xu Q. Zhang Z. Jiang L. Huang H. (2018). Efficient production of lycopene by engineered *E. coli* strains harboring different types of plasmids. Bioprocess Biosyst. Eng. 41, 489–499. doi: 10.1007/s00449-017-1883-y, PMID: 29313097

[ref91] Yang Q. (2021). Crucial roles of carotenoids as bacterial endogenous defense system for bacterial radioresistance of *Deinococcus radiodurans*. bioRxiv:2021.05.26.445811. doi: 10.1101/2021.05.26.445811

[ref92] Yu H. Cao L. Li F. Wu Q. Li Q. Wang S.. (2015). The antioxidant mechanism of nitroxide TEMPO: scavenging with glutathionyl radicals. RSC Adv. 5, 63655–63661. doi: 10.1039/C5RA06129F

[ref93] Zhang L. Yang Q. Luo X. Fang C. Zhang Q. Tang Y. (2007). Knockout of *crtB* or *crtI* gene blocks the carotenoid biosynthetic pathway in *Deinococcus radiodurans* R1 and influences its resistance to oxidative DNA-damaging agents due to change of free radicals scavenging ability. Arch. Microbiol. 188, 411–419. doi: 10.1007/s00203-007-0262-5, PMID: 17541775

[ref94] Zhang S. Zhao X. Tao Y. Lou C. (2015). A novel approach for metabolic pathway optimization: oligo-linker mediated assembly (OLMA) method. J. Biol. Eng. 9:23. doi: 10.1186/s13036-015-0021-0, PMID: 26702298 PMC4688952

[ref95] Zhou Z. Zhang W. Su S. Chen M. Lu W. Lin M.. (2015). CYP287A1 is a carotenoid 2-β-hydroxylase required for deinoxanthin biosynthesis in *Deinococcus radiodurans* R1. Appl. Microbiol. Biotechnol. 99, 10539–10546. doi: 10.1007/s00253-015-6910-9, PMID: 26311221

